# First Glance at Myeloid Leukaemia Factor 2 in Cardiomyocytes

**DOI:** 10.3390/jcdd13010019

**Published:** 2025-12-30

**Authors:** Jakob Christoph Voran, Lucia Sophie Kilian, Simone Martini, Marcin Luzarowski, Marie Isabel Noormalal, Oliver Josef Müller, Ashraf Yusuf Rangrez, Derk Frank

**Affiliations:** 1 Department of Internal Medicine III, Cardiology and Critical Care, University Hospital Schleswig-Holstein, Campus Kiel, 24105 Kiel, Germany; jakob.voran@uksh.de (J.C.V.); simone.martini@uksh.de (S.M.);; 2 German Centre for Cardiovascular Research (DZHK), Partner Site Hamburg/Kiel/Lübeck, 24105 Kiel, Germany; 3 Core Facility for Mass Spectrometry and Proteomics, Center for Molecular Biology at Heidelberg University (ZMBH), 69120 Heidelberg, Germany; 4 Department of Internal Medicine V, Angiology, University Hospital Schleswig-Holstein, Campus Kiel, 24105 Kiel, Germany; 5 Department of Cardiology, Angiology and Pneumology, Universitätsklinikum Heidelberg, Im Neuenheimer Feld 410, 69120 Heidelberg, Germany; 6 German Centre for Cardiovascular Research, Partner Site Rhein-Neckar, Partner Site Heidelberg, 69120 Heidelberg, Germany

**Keywords:** myeloid leukaemia factor-2, cardiomyocyte, desmin-related myopathy, protein aggregates

## Abstract

Understanding the molecular mechanisms that maintain protein homeostasis in cardiomyocytes is fundamental for the development of causal therapies for heart failure. Chaperones, the ubiquitin–proteasome system and autophagy are major regulators of cardiac homeostasis and are crucial for cardiomyocyte function and survival. In this context, myeloid leukaemia factor 2 (MLF2) emerged as a candidate of interest, as we found it overrepresented in protein aggregates in the hearts of mouse models of desmin-related cardiomyopathies (DRM), and it has also been suggested to be associated with dilated cardiomyopathy (DCM). Here, we identified αB-crystallin (CryAB), among other proteins, as a potential interaction partner of MLF2. Functionally, MLF2 was significantly upregulated in mouse models of heart failure and in two in vitro models of cardiomyocyte hypertrophy, and its overexpression resulted in attenuation of pro-hypertrophic gene expression. Taken together, these findings provide initial evidence supporting a role for MLF2 in regulating protein homeostasis and in modulating hypertrophic signalling in cardiomyocytes.

## 1. Introduction

Cardiomyocytes become terminally differentiated shortly after birth and permanently exit the cell cycle. Throughout life, however, they remain exposed to substantial mechanical and metabolic stress, making tight control of protein homeostasis essential for maintaining cardiomyocyte function and survival [1]. Chaperones, the ubiquitin–proteasome system, and autophagy constitute the major regulators of cardiac protein quality control and proteostasis [2]. A deeper understanding of these processes is crucial for the development of new causal therapies for heart failure—a condition with high incidence and prevalence, considerable disease burden, and poor long-term survival [3,4].

Myeloid leukaemia factor 2 (MLF2) was first described in 1996 in a screen for genes related to MLF1 [5]. The MLF2 gene is located on the short arm of chromosome 12 and encodes a 248-amino-acid protein [5]. In contrast to the predominantly testis-restricted expression of MLF1, MLF2 is consistently and highly expressed across a wide range of tissues, including the myocardium [5]. Because of their strong sequence similarity (over 60%), MLF1 and MLF2 have frequently been studied in parallel [5]. Early research on both proteins focused largely on their roles in malignant tumours: MLF2 overexpression enhances survival of various tumour cell lines, whereas MLF2 downregulation reduces tumour initiation and metastasis [6,7]. The proposed signalling pathways involving MLF2 include PI3K–AKT, p53, and Wnt [6]. Moreover, Dave et al. demonstrated that hypoxia induces MLF2 upregulation in tumour cells and that MLF2 modulates nitric oxide synthase activity [6]. Huang et al. further showed that DDB1- and CUL4-associated factor 8 (DCAF8) directly binds MLF2, promoting its ubiquitination and accelerated degradation [8]. Conversely, MLF2 is stabilised by the deubiquitinating enzyme USP11, which removes ubiquitin chains from MLF2 and thereby prolongs its half-life [8].

Beyond its role in cancer biology, MLF2 has recently gained attention in cardiac research. A transcriptomic study identified MLF2 as a potential biomarker for dilated cardiomyopathy (DCM) [9]. In addition, members of the MLF protein family have been detected in protein aggregates characteristic of desmin-related cardiomyopathies (DRM) [10]. DRM comprises a group of genetically heterogeneous disorders in which proteins interacting with desmin become misfolded and accumulate within cardiac and skeletal muscle cells [11]. These diseases are marked by disrupted protein homeostasis and serve as valuable models for studying regulatory proteins that control protein quality in cardiomyocytes [11].

Despite these intriguing observations linking MLF2 to cancer biology, DRM and DCM, its specific role in cardiomyocytes remains poorly understood.

In the present study, we sought to clarify potential functions of MLF2 in cardiomyocytes. Specifically, we addressed the following questions:

(i) What is the MLF2 interactome, and is it consistent with a potential role in DRM or DCM?
(ii) Is MLF2 regulated in in vitro models of DRM?
(iii) Is MLF2 differentially regulated in models of maladaptive hypertrophy and heart failure and does it influence experimentally induced maladaptive hypertrophy in vitro?
(iv) Does MLF2 modulate MLF1 expression or vice versa?

## 2. Materials and Methods

### 2.1. Laser Microdissection and Mass-Spectrometry

Male mice from DesD7-TG mice [12], CryAB^R120G^-TG mice [12,13] and Myozap-TG mice [14,15] were sacrificed and a section from the left ventricle (midventricular) was prepared. Cardiac tissues were immediately snap-frozen in liquid nitrogen and stored at −70 °C until further use. Approximately 7 µm cryosections were placed on membrane-coated (1 µm) slides, stained with cresyl violet and air-dried. Cardiac areas with protein aggregates were isolated by laser microdissection and pressure catapulting (Palm Microbeam; P.A.L.M. GmbH, Bernried, Germany). Isolated tissue pieces were collected in the cap of a 0.5 mL reaction tube. From each sample, 0.5–4 × 10^6^ µm^2^ area of heart tissue were collected and stored at −80 °C.

Each sample was lysed with 20 μL of extraction buffer (50 mM triethylammonium bicarbonate (TEAB) pH 8.0, 0.1% SDS). Samples were then mixed 1:1 with Laemmli sample loading buffer, heated at 95 °C for 5 min and run on the 12% SDS PAGE (with 4% stacking gel) until proteins entered the stacking gel. The individual gel pieces with protein fractions were diced into small fragments (approx. 1 mm^3^), then de-stained and dehydrated. The samples were reduced (10 mM dithiothreitol, 56 °C, 60 min in 100 mM ammonium bicarbonate (ABC) buffer, pH 7.4), alkylated (55 mM iodoacetamide, room temperature, 30 min in 100 mM ABC) and subjected to trypsin digestion at 37 °C in 100 mM TEAB buffer, pH 8, overnight. The peptides were extracted from the gel pieces with increasing concentrations of acetonitrile (ACN) (60%, 100%) and pooled with the supernatant from the overnight digestion, dried in Speedvac, and re-suspended in HPLC loading buffer (3% ACN, 0.5% trifluoroacetic acid). Chromatographic separation on a Dionex U3000 UHPLC system and further processing was performed as detailed elsewhere [16].

### 2.2. Yeast Two-Hybrid (Y2H)-Screen

The Y2H screen (ULTImate Y2H SCREEN, Hybrigenics Services SAS, Evry-Courcouronens, France) was performed by Hybrigenics Corp. with human MLF2 (aa 1-248) on the company’s human ventricle and embryo heart library. Two fusions were used: LexA-bait and bait-LexA.

### 2.3. Isolation and Culture of Neonatal Rat Cardiac Myocytes

For the preparation of neonatal rat ventricular cardiomyocytes (NRVCMs), male and female Wistar rat pups (Charles River) aged 1–2 days were sacrificed by decapitation. Hearts were extracted and transferred into ADS buffer (120 mmol/L NaCl, 20 mmol/L HEPES, 8 mmol/L NaH_2_PO_4_, 6 mmol/L glucose, 5 mmol/L KCl, and 0.8 mmol/L MgSO_4_; pH 7.4). Ventricles were isolated, minced, and digested in collagenase type II (0.5 mg/mL, Worthington Biochemical Corporation, Lakewood, NJ, USA) and pancreatin (0.6 mg/mL, Sigma-Aldrich, St. Louis, MO, USA) in sterile ADS buffer at 37 °C in four to five sequential digestion cycles until sufficient cell dissociation was achieved. The cell suspension was subsequently separated on a Percoll gradient (GE Healthcare, Chicago, IL, USA) to enrich cardiomyocytes and remove fibroblasts. Cardiomyocytes were plated in DMEM supplemented with 10% (*v*/*v*) foetal calf serum, 100 U/mL penicillin, 100 µg/mL streptomycin, and 2 mmol/L L-glutamine (Life Technologies, Carlsbad, CA, USA) for 24 h. After washing with warm PBS, cells were infected with adenoviral (Ad) vectors in serum-free medium. For treatment experiments, the medium was replaced after 48 h and cells were exposed to 100 µM phenylephrine (PE) for 48 h, or 50 nM Bafilomycin A1 (BfA) or 10 µM MG-132 (Sigma-Aldrich, St. Louis, MO, USA) for 24 h, respectively.

For stretching experiments, NRVCMs were plated on collagen type I-coated BioFlex^®^ membranes (Dunn Labortechnik, Asbach, Germany) at a density of 1.5 × 10^6^ cells per well. After 24 h, cells were washed with PBS, incubated in serum-reduced DMEM for an additional 24 h, and then transferred to the Flexlink FX5000T-FLK-Stretcher (Dunn Labortechnik) in fresh medium. Biaxial stretch (elongation factor 1.12) was applied for 48 h. Control NRVCM were plated on identical silicone membranes and underwent the same washing and media-change steps without stretch stimulation.

### 2.4. Isolation of Adult Rat Cardiac Myocytes (ARVCM)

For ARVCM isolation, six-week-old Wistar rats were used. The animals were anaesthetised with isoflurane, and 20 IU heparin was injected into the inferior vena cava. After removal of the thymus, the transverse aorta and pulmonary vessels were dissected, and the heart was cooled with ice-cold PBS. The coronary arteries were flushed with isolation buffer (120.4 mM NaCl, 14.7 mM KCl, 0.6 mM KH_2_PO_4_, 0.6 mM NaH_2_PO_4_·H_2_O, 1.2 mM MgSO_4_·7H_2_O, 10 mM HEPES, 4.6 mM NaHCO_3_, 30 mM taurine, 10 mM 2,3-butanedione monoxime, 5.5 mM glucose). Next, hearts were mounted on a Langendorff perfusion system (Bochem Instrumente, Weilburg, Germany) and perfused for 20 min at 8 mL/min with a digestion solution (50 mL isolation buffer containing 90 mg collagenase II (Worthington, 260–290 U/mg) and 40 µM CaCl_2_), which was continuously recirculated. After digestion, the aorta, atria, and right ventricle were removed. Enzymatic digestion was stopped by adding 5 mL stop buffer (isolation buffer supplemented with 1% (*w*/*v*) BSA and 12.5 µM CaCl_2_) and cells were suspended by pipetting up and down for 2–3 min until fully dissociated. The resulting cell suspension was filtered through a 200-µm mesh into a 50 mL tube.

### 2.5. Coimmunoprecipitation

Neonatal rat ventricular cardiomyocytes (NRVCMs, 10 × 10^6^ cells) were cultured in 10 cm dishes, harvested, and lysed in RIPA buffer supplemented with 1% SDS, protease inhibitor cocktail (Roche, Basel, Switzerland), and phosphatase inhibitor cocktails 2 and 3 (Sigma-Aldrich). Lysates were cleared of cellular debris by centrifugation, and protein concentrations were determined using the DC Protein Assay (Bio-Rad, Hercules, CA, USA). For co-immunoprecipitation, 250 µg of protein per condition was incubated with Dynabeads Protein G (Invitrogen, #10007D) coupled to MLF2 antibody (MLF2 (B-6), IgG2b, Santa Cruz, sc-166874, monoclonal, mouse) or with uncoupled beads as the negative control. Unbound proteins were removed in five washing steps, and bound proteins were eluted in 200 mM Tris, 8% SDS, 2% β-mercaptoethanol for 5 min at 95 °C. Dynabeads were removed using a magnetic rack, and the supernatant containing immunoprecipitated proteins was collected in a fresh tube for downstream analysis.

### 2.6. LC-Tandem Mass Spectrometry Analysis

Protein samples were prepared for digestion using the Single Tube Solid Phase Sample Preparation (SP3) method on a KingFisher Apex platform [17,18]. To ensure optimal results, all reagents, solutions, and vessels were of high purity and keratin-free. Magnetic bead-based sample preparation was carried out in a 96-well plate format using a prototype magnetic bead slurry (Promega, Cat. No. CS3325A04, Madison, WI, USA) and a total of 1000 µL liquid per sample for binding. Beads were washed three times with dH_2_O and reconstituted to a final concentration of 100 µg/µL. Protein reduction and alkylation were performed in the presence of 10 mM TCEP, 40 mM CAA, and 1% SDS. Samples were incubated at 95 °C for 5 min, followed by 25 min at 70 °C with mixing, and then cooled to room temperature. Following reduction and alkylation, 2 µL of magnetic bead slurry was added to each sample, followed by the addition of ethanol to a final concentration of 80%. Samples were incubated at 24 °C for 20 min with mixing to enable protein binding. Beads were subsequently washed three times with 1 mL of 80% ethanol and once with 1 mL of 80% acetonitrile (ACN), with each wash lasting 4 min and including mixing. Protein digestion was performed with trypsin at a 1:50 enzyme-to-protein ratio. The reaction was carried out at 37 °C for 4 h with mixing. Digestion was quenched by the addition of trifluoroacetic acid (TFA). On the following day, the pH of each sample was confirmed to be below 2 before proceeding to StageTip desalting and LC-MS analysis. Digested peptides were desalted using self-assembled C18 Empore^®^ extraction discs (3M, Maplewood, MN, USA) [19].

Samples were suspended in 0.1% TFA and analysed using an Ultimate 3000 liquid chromatography system coupled to an Orbitrap QE HF (Thermo Fisher, Waltham, MA, USA) as described previously [20]. Briefly, peptides were separated in a 60 min linear gradient starting from 3% B and increasing to 23% B over 50 min and to 38% B over 10 min, followed by washout with 95% B. The mass spectrometer was operated in data-dependent acquisition mode, automatically switching between MS and MS2. MS spectra (*m*/*z* 400–1600) were acquired in the Orbitrap at 60,000 (*m*/*z* 400) resolutions and MS2 spectra were generated for up to 15 precursors with a normalised collision energy of 27 and an isolation width of 1.4 *m*/*z*. The tandem mass spectrometry spectra were searched against the SwissProt Rattus norvegicus (UP000002494, November 2019) protein database and a customised contaminant database (part of MaxQuant, MPI Martinsried) using Proteome Discoverer 2.5 (https://www.thermofisher.com/order/catalog/product/OPTON-31099, November 2019) with Sequest HT (Thermo Fisher Scientific). A fragment ion mass tolerance was set to 0.02 Da and a parent ion mass tolerance to 5 ppm. Trypsin was specified as the enzyme. Carbamidomethylation was set as a fixed modification of cysteine, and oxidation (methionine) and deamidation (asparagine, glutamine) as variable modifications of peptides. Acetylation, methionine loss, and the combination of acetylation and methionine loss were set as variable modifications of the protein terminus. Peptide quantification was performed using the precursor ion quantifier node with the Top N Average (*n* = 3) method set for protein abundance calculation. Only proteins identified with at least two peptides and assigned as master proteins were used for the analysis. Protein intensities were normalised to the samples’ median. Protein quantification in at least one sample group (*n* = 2) was used as a further filtering criterion. Missing data was imputed (*n* = 2) using random draws from a manually defined left-shifted Gaussian distribution. Next, the ratio between the corresponding IP and noIP samples were calculated. The ggplot R package (R version 4.4.1 (2024-06-14 ucrt)) was employed for data visualisation. Additionally, we used ShinyGo for pathway analysis [21].

### 2.7. RNA Isolation and Purification, Reverse Transcription, and Quantitative Real-Time PCR

Total RNA was isolated from NRVCM using the Quick-RNA™ Microprep kit (Zymo research, Irvine, CA, USA) and from cardiac tissue using the QIAzol kit (Qiagen, Hilden, Germany) according to the manufacturer’s instructions. After DNase I (Thermo Fisher Scientific) digestion RNA concentration was measured with a Nanodrop spectrophotometer (Thermo Fisher Scientific). cDNA was synthesised from 1 μg DNA-free total RNA using a hexanucleotide random-primer-mix (Carl Roth, Karlsruhe, Germany) and the LunaScript^TM^ RT SuperMix kit (New England Biolabs, Ipswich, MA, USA). For quantitative real-time PCR (qRT-PCR) 10 ng cDNA was used with EXPRESS SYBR Green qPCR SuperMix Universal reagent (Life Technologies) or BioRad iQ Multiplex Powermix for Multiplex-PCR in a CFX96 Real-Time PCR detection system (Bio-Rad).

### 2.8. Cloning of Rat Mlf2 and the Synthetic miRNA for Mlf2-Knockdown

Mlf2 overexpression (Ad-MLF2) and knockdown (Ad-miR-MLF2) constructs were generated using the Gateway^®^ cloning system (Thermo Fisher Scientific) following the manufacturer’s protocols. In brief, rat Mlf2 cDNA was first cloned into the pDONR221 entry vector via BP clonase and subsequently recombined into the pAd/CMV/V5-DEST destination vector using LR clonase (Thermo Fisher Scientific). For Mlf2 knockdown, synthetic microRNA (miRNA) oligonucleotides targeting rat Mlf2 were designed. The annealed oligonucleotides were cloned into pcDNA6.2-GW/miR using the Block-iT^®^ Pol II miR RNAi Expression Vector Kit and subsequently recombined into the pAd/CMV/V5-DEST vector as described above. Positive clones were digested with PacI, and the resulting DNA fragments were transfected into HEK293A cells. After several days, cells were harvested, and adenoviral particles were isolated using freeze–thaw cycles followed by centrifugation. Primers used for cloning rat Mlf2 cDNA: Forward: Mlf2_gw_F (5′-gct ggc acc gcc acc atg ttc cgc ttc atg agg-3′), Reverse: Mlf2_gw_R (5′-gct ggg tcg cc tca cca gtc ata gcg acg gg-3′). Oligonucleotides used for generating rat-specific synthetic miRNA for Mlf2 knockdown: Top strand: 5′-tgc tga gga cag tca ttt caa gga ttg ttt tgg cca ctg act gac aat cct tgatga ctg tcc t-3′, Bottom strand: 5′-cct gag gac agt cat caa gga ttg tca gtc agt ggc caa aac aat cct tga aat gac tgt cct c-3′.

### 2.9. Mlf1 Overexpression and Mlf1-Knockdown

Mlf1 was manipulated as previously described [22]. In brief, we used the same method as detailed for Mlf2 with the following primers: Mlf1 rat cDNA = Mlf1_gw_F (5′ gct ggc acc atg ttc cgg atg ttg agc 3′) and Mlf1_gw_R (5′ gct ggg tcg cct tat ttt ttg gtg att ttc 3′). Oligonucleotides for the synthetic miRNA (knockdown): top strand (5′ tgc tga aga ggt tca gag aaa ctt ctg ttt tgg cca ctg act gac aga agt ttc tga acc tct t 3′) and bottom strand (5′ cct gaa gag gtt cag aaa ctt ctg tca gtc agt ggc caa aac aga agt ttc tct gaa cct ctt c 3′).

### 2.10. Protein Isolation and Immunoblotting

NRVCMs were harvested using RIPA buffer supplemented with a protease inhibitor cocktail (Roche) and phosphatase inhibitor cocktails 2 and 3 (Sigma-Aldrich), followed by three rapid freeze–thaw cycles. Protein concentrations of the lysates were determined using the DC Protein Assay Kit (Bio-Rad), after which samples were normalised to equal protein amounts and subjected to SDS–PAGE, followed by transfer to a nitrocellulose membrane (GE Healthcare). Membranes were blocked and incubated with primary antibodies overnight at 4 °C. After three washes in 0.1% TBST, membranes were incubated with the appropriate HRP- or Cy3-conjugated secondary antibodies for 2 h, followed by three additional wash steps. For HRP-based detection, proteins were visualised using ECL Select Western blotting Detection Reagents (GE Healthcare). All antibodies used in this study are listed in Supplementary Materials Table S1. Densitometric quantification was performed using ImageJ.

### 2.11. Immunofluorescence Microscopy

For immunofluorescence microscopy, NRVCMs were seeded on collagen-coated glass coverslips (BD Biosciences, Franklin Lakes, NJ, USA) and infected with the respective viruses as required. Cells were fixed with 4% (*w*/*v*) paraformaldehyde (PFA; Sigma-Aldrich) in PBS for 5 min at room temperature, followed by permeabilization with 0.1% (*v*/*v*) Triton X-100 (Sigma-Aldrich) and blocking with 2.5% (*w*/*v*) BSA in PBS for 1 h at room temperature. Primary fluorophore-conjugated secondary antibodies were diluted in 2.5% (*w*/*v*) BSA in PBS and incubated sequentially for 1 h (primary antibodies) and 2 h (secondary antibodies) at room temperature. Nuclei were counterstained with DAPI (1 μg/mL; Sigma-Aldrich) during the secondary antibody incubation. Coverslips were mounted using FluorPreserve mounting medium (Calbiochem, Darmstadt, Germany). Images were acquired using a ZEISS LSM 800 confocal laser scanning microscope and processed with ZEN software (Zeiss, Oberkochen, Germany).

### 2.12. Animal Experiments

All experiments were approved by the Ministry of Energy Transition, Climate Protection, Environment, and Nature and were performed strictly following the legal and ethics guidelines from the EU Directive 2010/63/EU and according to ARRIVE-guidelines. Male animals aged 10 to 12 weeks were used for all experiments with mice.

RNA-Samples from Calcineurin-TG (Cn-TG) mice were compared to wild-type controls. Cn-TG mice (C57BL/6-Tg(α-MHC-Ppp3ca)37Eno/0) express a constitutively active form of calcineurin under the α-MHC promoter specifically in the heart [23].

Samples for the transverse aortic constriction (TAC) experiment were obtained from existing stocks of αMHC-Cre C57BL/6N mice from a previous study. Sample size was calculated using G*Power 3 [24], assuming a 1.5-fold biologically meaningful difference and an expected higher standard deviation in the TAC group. The TAC procedure was performed as follows: Mice were anaesthetised with isoflurane (4% for induction, 3% for maintenance) in combination with buprenorphine (0.1 mg/kg body weight, subcutaneously). During surgery, animals were ventilated via a 22-gauge endotracheal tube (Harvard Apparatus, Holliston, MA, USA) at 120 breaths/min (0.2 mL tidal volume). A lateral thoracotomy was used to access the aortic arch. TAC was induced between the brachiocephalic and left carotid arteries by tying a Prolene 6-0 suture around the aorta using a 27-gauge needle as a spacer. Sham-operated mice underwent the same procedure without ligation. Animals were randomly assigned to experimental groups; investigators were not blinded to group allocation. Only mice with echocardiographically confirmed TAC were included in the analysis. Cardiac tissue was collected 14 days after TAC.

### 2.13. Statistical Analysis

All data are presented as the mean ± the standard deviation, unless stated otherwise. Statistical comparisons between groups were performed using two-tailed Student’s *t*-test, one-way-ANOVA or two-way-ANOVA (as stated) in the software GraphPad PRISM (10.6.1). The significance level was set at *p* < 0.05.

## 3. Results

### 3.1. MLF2 Is Enriched in Protein Aggregates in DesD7-, CryAB^R120G^-, and Myozap-TG Mouse Hearts

To investigate general mechanisms in proteostasis across different cardiomyopathy models, we examined two established models of desmin-related cardiomyopathy (DesD7-TG and CryAB^R120G^-TG [12,13]) together with Myozap-TG mice, which we have previously shown to develop a phenotype characterised by protein aggregation and cardiomyopathy, independent of desmin-related pathology [14,15]. Protein aggregates were isolated by laser microdissection and subjected to LC–MS/MS analysis. Comparative proteomics revealed that MLF2 and MLF1 were consistently enriched in aggregates from all three models (Supplementary Table S2). Given that MLF1 has been examined previously in the context of mechanosensitive signalling, whereas the role of MLF2 in cardiomyocytes remains largely unexplored, we focused subsequent analyses on MLF2.

### 3.2. Interaction Partners of MLF2

To identify interaction partners of MLF2, a yeast two-hybrid (Y2H) screen was performed using cDNA libraries from human adult and embryonic ventricular myocardium. Among 208 million screened interactions, 30 positive clones were analysed in detail. CryAB emerged as a very high-confidence interactor, and HERPUD1 was identified with high confidence (Table 1).

**Figure 1:**
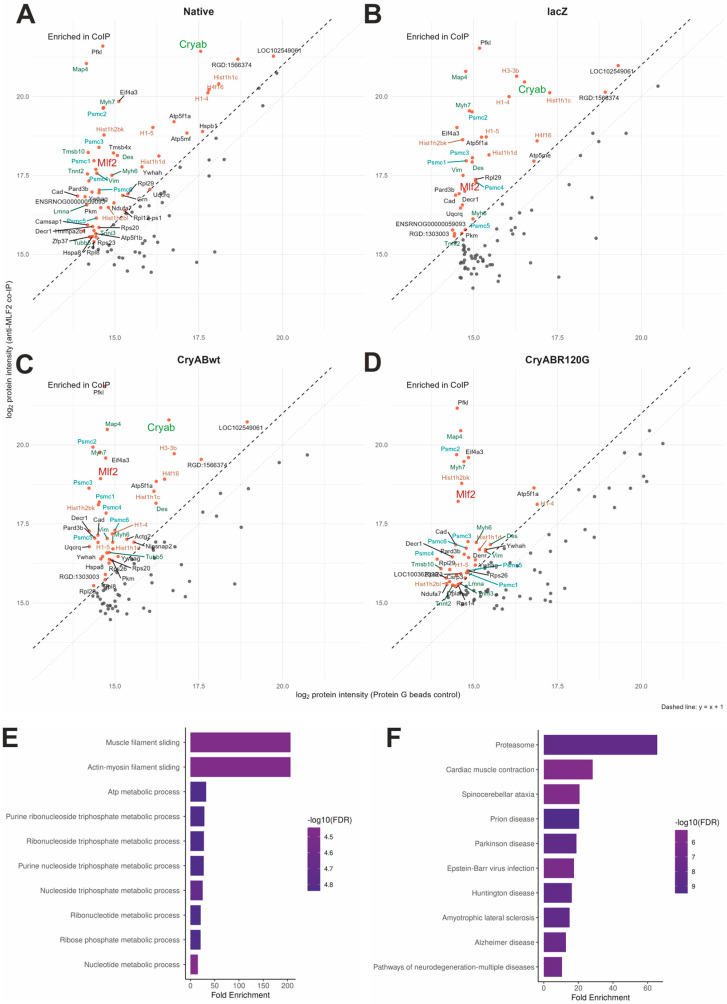
Identification of MLF2 interaction partners. (**A**–**D**) NRVCM (**A**) and NRVCM overexpressing lacZ (**B**), CryAB^wt^-tGFP (**C**), and expressing CryAB^R120G^-tGFP (**D**) were cultured and subjected to Co-IP of MLF2 (*n* = 4 per condition). After washing and elution, co-precipitated proteins were analysed by LC-MS/MS. Protein G beads without a coupled antibody served as negative controls. (**A**–**D**) Enriched proteins highlighting proteasome subunits (PSMC1–6, blue), histones (brown), cytoskeleton- and structure-related proteins (dark green), and CryAB (light green). (**E**) Gene Ontology (GO) terms associated with enriched proteins. (**F**) KEGG pathways associated with enriched proteins.

To complement these findings, we conducted co-immunoprecipitation (Co-IP) using anti-MLF2 antibodies in NRVCM under four conditions (native cells, LacZ overexpression, CryAB^WT^-tGFP, and CryAB^R120G^-tGFP), followed by LC–MS/MS analysis. Across all three control conditions (native cells, LacZ overexpression and CryAB^WT^-tGFP) 52 proteins were enriched in the MLF2 pull-down (Figure 1A–C). Among these, CryAB was the only protein detected in both the Y2H and Co-IP datasets. Additional enriched proteins included proteasomal components (PSMC1–6), histones, cytoskeletal and structural proteins (DES, LMNA, MYH6/7, TUBB, MAP4, TNNT2, VIM, TMSB10), members of the 14-3-3 family (YWHAG, YWHAH), and metabolic enzymes (UQCRQ, PKM, PFKL). Corresponding GO (Figure 1E) and KEGG (Figure 1F) annotations highlighted pathways associated with DNA interaction, proteasomal and metabolic processes, and cardiomyocyte contractility. Notably, CryAB was not enriched in the CryAB^R120G^-tGFP condition, indicating that the pathogenic mutation disrupts the interaction with MLF2 (Figure 1D).

### 3.3. Subcellular Localization of MLF2 in Cardiomyocytes

**Figure 2:**
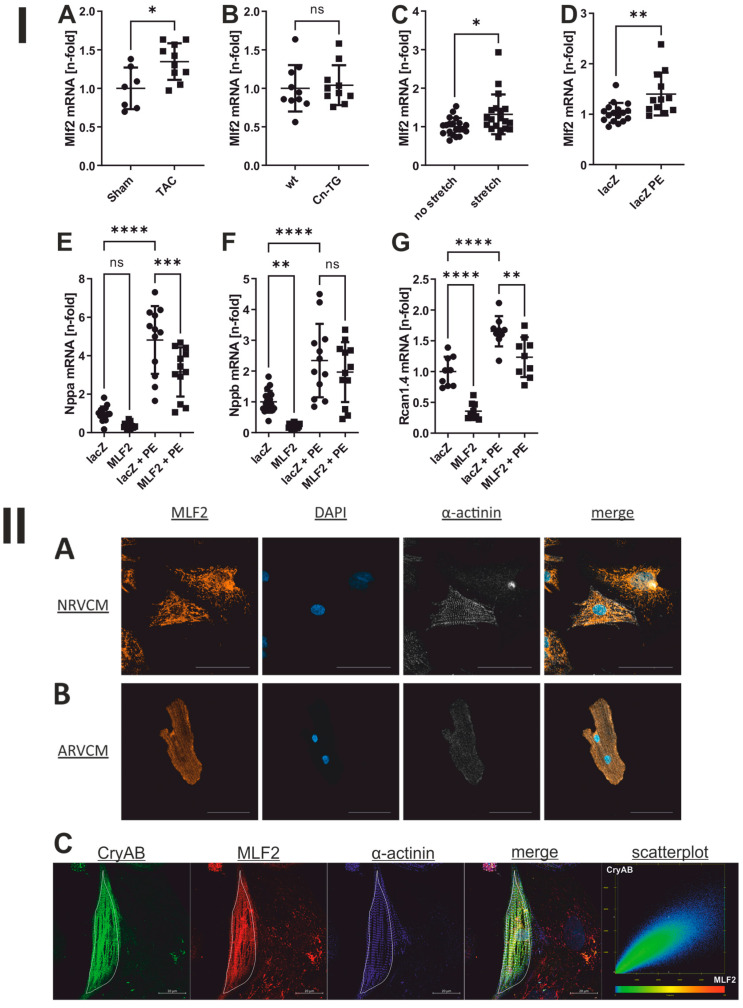
**Panel I**: MLF2 in hypertrophy. **I**(**A**–**D**) Mlf2 mRNA expression in different hypertrophy models: **I**(**A**) αMHC-Cre mice after TAC or sham surgery, **I**(**B**) calcineurin-transgenic mice (Cn-TG) versus wild type (WT) controls, **I**(**C**) NRVCM subjected to biomechanical stretch versus static controls, and **I**(**D**) NRVCM infected with lacZ and stimulated with PE versus unstimulated controls. **I**(**E**–**G**) Effect of MLF2 overexpression on PE-induced hypertrophic gene expression in NRVCM, shown for Nppa **I**(**E**), Nppb **I**(**F**), and Rcan1.4 **I**(**G**). *n* = 7–19. Statistical analysis using two-tailed Student’s t-test **I**(**A**–**D**) and two-way-ANOVA **I**(**E**–**G**). * *p* < 0.05, ** *p* < 0.01, *** *p* < 0.001, **** *p* < 0.0001, ns: not significant. **Panel II**: Subcellular localisation of MLF2 and colocalisation with CryAB **II**(**A**,**B**) Representative immunofluorescence staining of NRVCM **II**(**A**) and ARVCM **II**(**B**). Pseudocolours: MLF2 (orange), nuclei (DAPI, blue), Sarcomeric α-actinin (white). Scale bar = 50 µm. **II**(**C**) Colocalisation of CryAB and MLF2 in NRVCM. Pseudocolours: CryAB (green), MLF2 (red), Sarcomeric α-actinin (purple). Scale bar = 20 µm.

To determine the intracellular localization of MLF2, we analysed neonatal and adult rat ventricular cardiomyocytes by immunofluorescence. In NRVCM, MLF2 displayed a perinuclear, reticular distribution with extensions into the cell periphery (Figure 2II(A)), whereas in adult cardiomyocytes it aligned predominantly along the longitudinal axis of the cell (Figure 2II(B)). To further confirm the potential interaction with CryAB as indicated by both the Y2H-Screen and the Co-IP with LC-MS/MS analysis, we performed colocalization experiments in NRVCM, confirming substantial spatial overlap between MLF2 and CryAB (Figure 2II(C)).

### 3.4. MLF2 Expression in Models of Maladaptive Hypertrophy and Heart Failure

To assess whether MLF2 is regulated during cardiac stress, we analysed its expression in established in vivo and in vitro hypertrophy models. In a mouse model for induced pressure overload (TAC), MLF2 RNA and protein levels in the left ventricle were increased compared with sham controls (*p* = 0.0131) (Figure 2I(A) and Supplementary Figure S3). In calcineurin-transgenic mice (Cn-TG), a mouse model for pathological cardiac hypertrophy, MLF2 mRNA expression did not differ from wild-type hearts (1.0 vs. 1.041, *p* = 0.7), whereas protein levels were elevated (Figure 2I(B) and Supplementary Figure S3).

In NRVCM exposed to biomechanical load by biaxial stretch, MLF2 expression increased 1.3-fold (*p* = 0.0155) (Figure 2I(C)). Stimulation with phenylephrine (PE), representing neurohumoral hypertrophic stress, resulted in a 1.4-fold increase (*p* = 0.0037) (Figure 2I(D)). In summary, MLF2 expression was upregulated in both in vivo and in vitro models of maladaptive hypertrophy.

### 3.5. Influence of MLF2 on PE-Induced Hypertrophic Signalling

To determine whether MLF2 modulates the hypertrophic gene programme, we overexpressed MLF2 in NRVCM using an adenoviral vector (Ad-MLF2), with Ad-lacZ as the control. Even under basal conditions, MLF2 overexpression reduced the expression of the hypertrophy-associated genes *Nppa*, *Nppb*, and *Rcan1.4* (Ad-lacZ vs. Ad-MLF2: *Nppa* 1.0 ± 0.35 vs. 0.3 ± 0.15, *p* = 0.27; *Nppb* 1.0 ± 0.35 vs. 0.2 ± 0.07, *p* = 0.0075; *Rcan1.4* 1.0 ± 0.24 vs. 0.36 ± 0.14, *p* < 0.001; Figure 2I(E–G)).

MLF2 overexpression also attenuated the induction of hypertrophic markers following PE stimulation (*Nppa* 4.8 ± 1.76 vs. 3.2 ± 1.28, *p* = 0.0008; *Nppb* 2.3 ± 1.19 vs. 1.97 ± 0.97, *p* = 0.57; *Rcan1.4* 1.6 ± 0.24 vs. 1.2 ± 0.3, *p* = 0.005; Figure 2I(E–G)).

### 3.6. Reciprocal Regulation of MLF1 and MLF2

**Figure 3:**
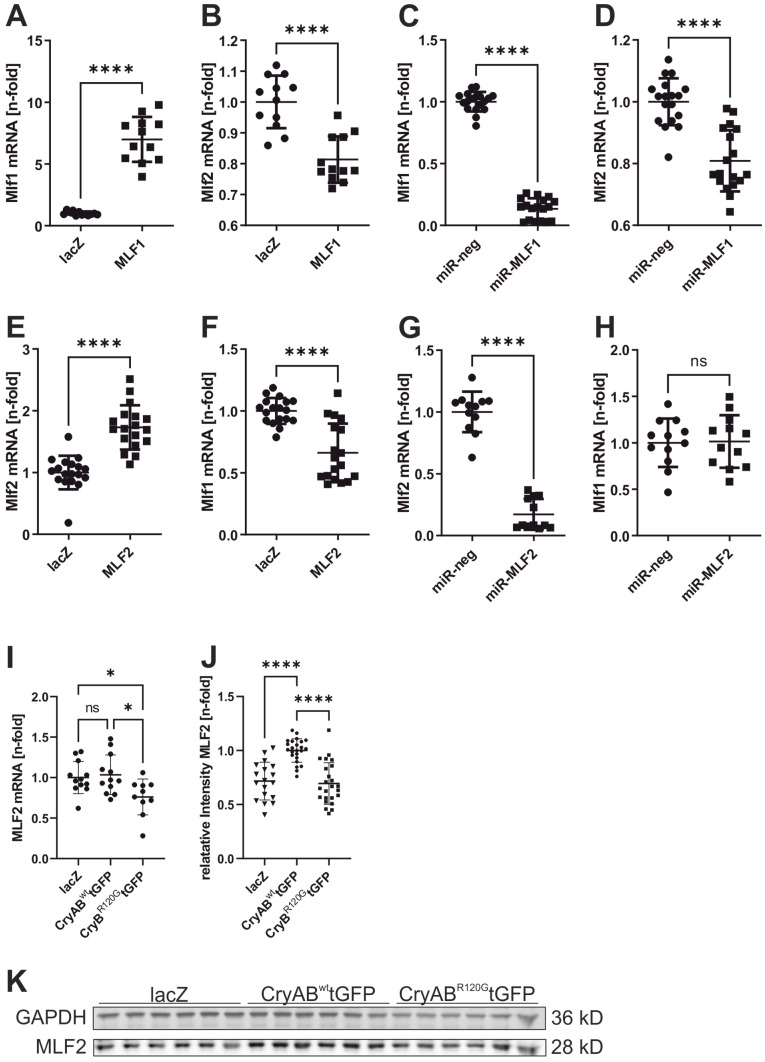
Reciprocal regulation of Mlf1 and Mlf2 expression. NRVCM were infected with adenoviral vectors for overexpression or miRNA-mediated knockdown of *Mlf1* or *Mlf2.* LacZ and miR-neg served as controls. Upper row: *Mlf1* overexpression (**A**) and its effect on *Mlf2* expression (**B**); *Mlf1* knockdown (**C**) and corresponding effects on *Mlf2* expression (**D**). Lower row: *Mlf2* overexpression (**E**) and effects on *Mlf1* expression (**F**); *Mlf2* knockdown (**G**) and corresponding effects on *Mlf1* expression (**H**). *n* = 12–18. Statistical analysis: two-tailed Student’s *t*-test. * *p* < 0.05, **** *p* < 0.0001, ns: not significant. (**I**) *Mlf2* mRNA expression following overexpression of lacZ, CryAB^wt^-tGFP, or CryAB^R120G^-tGFP. (**J**,**K**) Western blot analysis of MLF2 protein levels after overexpression of lacZ, CryAB^wt^-tGFP, or CryAB^R120G^-tGFP. *n* = 10–24. Statistical analysis: one-way ANOVA. * *p* < 0.05, **** *p* < 0.0001, ns: not significant.

Given the high sequence similarity between MLF2 and MLF1, we examined whether the two proteins compensate for or regulate one another. NRVCM were subjected to overexpression or knockdown of either MLF protein (Figure 3A–H). MLF1 overexpression significantly reduced MLF2 expression (Ad-lacZ vs. Ad-MLF1: 1.0 ± 0.07 vs. 0.81 ± 0.08, *p* < 0.0001; Figure 3B). Surprisingly, MLF1 knockdown also decreased MLF2 levels (Ad-miR-neg vs. Ad-miR-MLF1: 1.0 ± 0.08 vs. 0.8 ± 0.09, *p* < 0.0001; Figure 3D). Conversely, MLF2 overexpression reduced MLF1 expression (Ad-lacZ vs. Ad-MLF2: 1.0 ± 0.1 vs. 0.66 ± 0.24, *p* < 0.0001; Figure 3F), whereas MLF2 knockdown had no measurable effect on MLF1 (Figure 3H).

### 3.7. Regulation of MLF2 Expression by Wild-Type and Mutant CryAB

To evaluate whether the disease-causing CryAB^R120G^ mutation affects MLF2 expression, we overexpressed CryAB^WT^-tGFP or CryAB^R120G^-tGFP in NRVCM, an established cell-culture model to induce DRM in vitro [25,26]. CryAB^WT^-tGFP overexpression increased MLF2 protein levels, whereas the R120G mutant did not (Ad-lacZ vs. Ad-CryAB^WT^-tGFP vs. Ad-CryAB^R120G^-tGFP: 0.68 ± 0.18 vs. 1.0 ± 0.09 vs. 0.72 ± 0.2, *p* < 0.0001; Figure 3J).

MLF2 mRNA levels were unchanged by CryAB^WT^-tGFP but decreased following CryAB^R120G^-tGFP expression (Figure 3I), suggesting that wild-type CryAB stabilises MLF2 post-transcriptionally, whereas the mutant may impair this regulation.

### 3.8. MLF2 and Proteostatic Degradation Pathways

Given the association of MLF2 with protein aggregates and the interaction with CryAB, we assessed whether MLF2 is influenced by, or contributes to, autophagy, ubiquitination, or the unfolded protein response (UPR).

**Figure 4:**
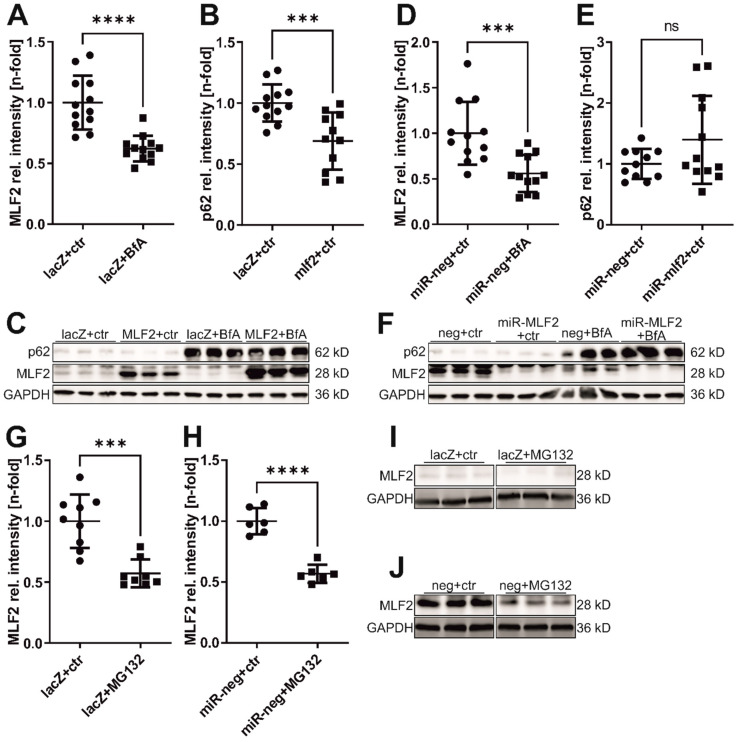
MLF2 in autophagy and proteasome regulation. NRVCMs were treated with Bafilomycin (BfA) or MG132 and infected with Ad-MLF2, Ad-miR-MLF2, or respective controls. (**A**,**D**) Effects of BfA treatment on MLF2 protein levels. (**B**,**E**) p62 protein levels following *Mlf2* overexpression or knockdown. Representative Western blots are shown in (**C**,**F**). (**G**–**J**) Effects of MG132 treatment on MLF2 protein levels, with densitometric analyses shown in (**G**,**H**), and representative Western blots in (**I**,**J**). *n* = 6–12. Statistical analysis: two-tailed Student’s *t*-test. *** *p* < 0.001, **** *p* < 0.0001, ns = not significant.

Treatment of NRVCM with the autophagy inhibitor bafilomycin A1 (BfA) significantly decreased MLF2 protein expression (Ad-lacZ + ctr vs. Ad-lacZ + BfA: 1.0 vs. 0.62, *p* < 0.0001; Ad-miR-neg + ctr vs. Ad-miR-neg + BfA: 1.0 vs. 0.56, *p* = 0.0009; Figure 4A,C,D,F). MLF2 overexpression reduced levels of the autophagy marker p62, whereas MLF2 knockdown showed a not significant trend toward increased p62 (Ad-lacZ vs. Ad-MLF2: 1.0 vs. 0.69, *p* = 0.001; Ad-miR-neg vs. Ad-miR-MLF2: 1.0 vs. 1.4, *p* = 0.09; Figure 4B,C,E,F). No significant changes were observed in other autophagy-related proteins (BECN1, LAMP1, RAB7, LC3B; Supplementary Figure S1A–J).

Inhibition of the proteasome with MG132 also decreased MLF2 protein abundance (Ad-lacZ + ctr vs. Ad-lacZ + MG132: 1.0 vs. 0.57, *p* = 0.0002; Ad-miR-neg + ctr vs. Ad-miR-neg + MG132: 1.0 vs. 0.56, *p* < 0.0001; Figure 4G–J). MLF2 overexpression did not alter global ubiquitination, whereas MLF2 knockdown increased ubiquitinated proteins and free ubiquitin (Supplementary Figure S1K–N).

To determine whether MLF2 affects ER stress pathways, we analysed mRNA levels of UPR components (*tXbp1*, *usXbp1*, *sXbp1*, *Chop*, *Atf4*). Neither MLF2 overexpression nor knockdown significantly altered expression of these markers (Supplementary Figure S2).

## 4. Discussion

The starting point of our study was the identification of MLF2 as a component of protein aggregates in several mouse models characterised by pathological protein aggregation. MLF2 emerged as a particularly interesting candidate due to its close homology to MLF1—which we previously described as being associated with cardiomyocyte proliferation, hypertrophy, and apoptosis—and its robust expression in cardiomyocytes [5,22,27].

This initial observation aligns with findings from a proteomic analysis by Maerkens et al., which identified MLF2 as being overrepresented in protein aggregates derived from muscle biopsies of patients with desminopathy or filaminopathy, compared to unaffected cytoplasmic tissue from the same individuals [10]. In addition, MLF2 has been shown to colocalise to protein aggregates in other non-cardiac diseases and disease models with pathological protein deposition. In both human patients and mouse models carrying a C9orf72 mutation, leading to deposition of polyGA-inclusion bodies and a phenotype similar to amyotrophic lateral sclerosis with frontotemporal dementia, co-deposition of MLF2 with the polyGA deposits has been demonstrated [28] Similarly, in a Drosophila model of Huntington’s disease, spatial proximity of dMLF2 to toxic poly(Q) deposits has been shown and a protective role of MLF2 was suggested [29]. Consistent with this, recent studies in non-cardiac systems identified MLF2 as a key component of nuclear envelope–associated condensates. In Torsin-deficient cells, MLF2 accumulates within nuclear envelope blebs, where it interacts with chaperones such as HSP70 and DNAJB6 to regulate condensate formation and prevent aberrant protein aggregation [30,31]. Taken together, the consistent association of MLF2 with protein deposits across diverse diseases suggests a mechanistic involvement in underlying pathophysiological processes.

To explore the potential functional role of MLF2, we investigated its protein interaction network. Our analysis revealed interaction partners indicative of a role in protein quality control in cardiomyocytes, particularly in the context of desmin-related myopathy (DRM). In an initial Y2H screen, we identified two proteins with known functions in ER stress response and DRM—CryAB and HERPUD1—as candidate interaction partners of MLF2 [32,33]. Subsequent LC-MS/MS analysis confirmed CryAB and further identified proteasome subunits, histones, cytoskeletal components, 14-3-3 family members (further supported by the presence of a predicted 14-3-3 binding motif within the MLF2 amino acid sequence [34]) and energy metabolism-associated proteins (UQCRQ, PKM, PFKL) as part of the MLF2 interactome. Colocalization studies of MLF2 and CryAB revealed strong overlap in fluorescence signals, suggesting spatial proximity and possible functional interplay.

CryAB is a well-characterised small heat shock protein with regard to DRM, as it is directly involved in the folding of desmin and a point mutation leading to a single amino acid change in this chaperone protein, namely the CryAB^R120G^ mutation, leads to DRM [35,36]. In DRM, undegraded misfolded proteins form larger complexes, continue to assemble, and subsequently form protein aggregates [36]. At this stage, the aggregates either adopt parallel β-fold structures or continue to aggregate and form larger insoluble deposits that are transported retrogradely from the periphery to the nucleus via microtubules forming perinuclear aggresomes [36]. The protein deposits in β-fold structure form soluble pre-amyloid oligomers (PAO) and accumulate in the cytoplasm [36]. Pathomechanistically, the CryAB^R120G^ mutation leads to heart failure in several ways. On the one hand, the high cytoplasmic PAO concentration itself is cytotoxic. On the other hand, the protein degradation pathway is damaged in DRM, leading to mitochondrial dysfunction and an increase in apoptosis [37,38,39]. In addition to the role of the mutated CryAB in DRM, Kumarapeli et al. have shown that overexpression of non-mutated CryAB in mice suppresses maladaptive cardiac remodelling and decreases cardiac hypertrophy [40], demonstrating a protective function of CryAB in hypertrophy.

In our study, we overexpressed either wild-type CryAB or the pathogenic CryAB^R120G^ mutant in NRVCM. Interestingly, wild-type but not mutated CryAB^R120G^ led to a marked increase in MLF2 protein levels without affecting MLF2 mRNA expression, suggesting post-transcriptional stabilisation of MLF2 by wild-type CryAB, an effect that could not be observed after overexpression of mutated CryAB^R120G^, pointing to a lost interaction with MLF2 due to a possible conformation change in mutated CryAB^R120G^.

Consistent with this, we found no enrichment of CryAB protein in MLF2 co-immunoprecipitates after overexpression of CryAB^R120G^, whereas the protein was overrepresented in all three control conditions including the wild-type CryAB overexpression samples, suggesting that the disease-causing mutation impairs the interaction with MLF2. The loss of this interaction may represent a previously unrecognised component of the DRM pathomechanism.

Protein aggregation within the heart can contribute to cardiac pathologies, including the development of myocardial hypertrophy [41]. To investigate a potential influence of MLF2 on cardiac disorders, we further explored the role of MLF2 in cardiac disease models. MLF2 expression was significantly upregulated in vivo following pressure overload (TAC) and in vitro in NRVCM upon mechanical stretch or PE stimulation. These results are in line with data from Guo et al., who reported increased MLF2 levels in myocardial tissue from DCM patients [9]. Functional assays supported this distinction: overexpression of MLF2 reduced expression of hypertrophic markers (*Nppa*, *Nppb*, *Rcan1.4*) under both basal and PE-stimulated conditions. In contrast, MLF1 expression was found to be downregulated in in vivo models of cardiac hypertrophy induced by pressure overload or neurohumoral stimulation, as well as upon mechanical stretch in NRVCM [22]. Consistently, MLF1 expression has been found to be reduced in human DCM samples [22]. Additionally, MLF1 overexpression led to increased expression of pro-hypertrophic markers in settings comparable to ours [27]. These opposing effects suggest that MLF1 and MLF2 may have antagonistic roles in cardiac hypertrophy. Supporting this notion, we observed that MLF1 overexpression downregulated MLF2 expression, and vice versa, indicating an inverse regulation that may have functional relevance in the context of cardiac hypertrophy. That we observed no upregulation of the respective other factor following knockdown of either MLF1 or MLF2 may be explained by (over-)compensatory mechanisms that maintain (or even reduce) expression of the counterpart, as well as by residual protein present at very low levels, which may be sufficient to prevent RNA upregulation.

The presence of MLF2 within protein aggregates, together with its interactome profile, points toward a role in proteostasis. Within the scope of our project, we performed preliminary experiments assessing the relationship between MLF2 and autophagy, ubiquitination, and the unfolded protein response (UPR) in NRVCM, analysing the effects of MLF2 overexpression and downregulation on these protein degradation processes, and the effects of inhibition of these processes on MLF2 expression. Pharmacological inhibition of autophagy (by BfA) or proteasomal degradation (by MG132) led to a reduction in MLF2 protein levels, which may reflect a yet unexplored protective or adaptive response of the cell, potentially involving decreased MLF2 synthesis with as-yet uninvestigated beneficial effects. This effect is not observed upon MLF2 overexpression, as the artificially induced expression potentially overrides the cell’s ability to downregulate MLF2 synthesis, resulting in its accumulation. It is also possible that MLF2 is degraded via alternative pathways, such as secretion or cytosolic proteases independent of the proteasome, although these mechanisms remain to be investigated.

MLF2 overexpression resulted in decreased levels of the autophagy marker p62, without significantly affecting global ubiquitination. MLF2 knockdown, on the other hand, led to elevated ubiquitination and free ubiquitin accompanied by a modest, statistically not significant increase in p62 levels, a protein responsible for the sequestration of ubiquitinated proteins into aggregates, potentially reflecting the cell’s efforts to maintain proteostasis.

However, neither MLF2 overexpression, nor MLF2 downregulation had any effect on the protein levels of other autophagy markers (BECN1, LAMP1, RAB7, LC3B) nor the expression of the markers for ER stress (XBP1, CHOP and ATF4), suggesting that MLF2 may selectively modulate specific components of the proteostasis network rather than globally altering autophagy or UPR pathways. Further experiments are necessary to determine the role of MLF2 in cellular proteostasis.

In summary, our study provides first insights into the potential role of MLF2 in cardiomyocytes, highlighting its involvement in hypertrophic signalling and protein homeostasis. We show that MLF2, identified in protein aggregates across cardiac and non-cardiac disease models, interacts with proteins involved in proteostasis, including CryAB.

Overexpression of wild-type CryAB, but not the pathogenic CryAB^R120G^ mutant, led to increased MLF2 protein levels without affecting mRNA expression, suggesting post-transcriptional stabilisation, which becomes lost in the mutant CryAB. Supporting this hypothesis, co-immunoprecipitation also suggests that the CryAB^R120G^ mutation disrupts the interaction with MLF2, pointing to a potential novel component of the DRM pathomechanism.

We demonstrate that MLF2 is significantly upregulated in a mouse model of pressure overload–induced heart failure, as well as in two in vitro models of cardiomyocyte hypertrophy. Consistent with these findings, MLF2 overexpression reduced the expression of pro-hypertrophic gene markers in NRVCM under both basal conditions and following PE stimulation, suggesting a protective role in pathological cardiac remodelling. Additionally, we could show that MLF2 overexpression downregulates MLF1 expression, and vice versa. This reciprocal regulation is consistent with our previous observations that MLF1 promotes hypertrophic gene expression under similar conditions, highlighting the antagonistic roles of MLF1 and MLF2 during cardiac hypertrophy

The presence of MLF2 in protein aggregates and its interactome profile indicate a role in proteostasis. Although first experiments assessing autophagy, ubiquitination, and the UPR are preliminary and do not allow definitive conclusions regarding its functional role in proteostasis yet, our data offer a valuable starting point for deeper mechanistic investigations into MLF2 as a potential modulator of cardiomyocyte stress responses.

## 5. Conclusions

Our study identifies MLF2 as a novel component of protein aggregates in cardiomyocytes and highlights its potential role in the regulation of cardiac proteostasis and hypertrophic signalling. Across multiple mouse models of desmin-related cardiomyopathies, MLF2 was consistently enriched in protein aggregates and interacted with key proteins involved in protein quality control, including αB-crystallin (CryAB). Functional assays demonstrated that MLF2 overexpression attenuates pro-hypertrophic gene expression under both basal and stress conditions, suggesting a protective role in maladaptive cardiac remodelling. Furthermore, the reciprocal regulation between MLF2 and MLF1 supports the concept of antagonistic roles for these closely related proteins in cardiomyocyte hypertrophy. Preliminary analyses of MLF2 in autophagy, ubiquitination, and unfolded protein response pathways indicate a selective contribution to proteostasis, although further mechanistic studies are required. Collectively, our findings provide a foundation for future investigations into MLF2 as a modulator of cardiomyocyte stress responses.

## Figures and Tables

**Table 1:** MLF2 Protein Interaction Partners detected via Y2H Screening.

Gene Name	GenBank ID (NCBI)	PBS *	Number of Positive Clones
CRYAB var2	NM_001289807.1	A	12
HERPUD1	NM_014685.4	B	3

* PBS: Predicted Biological Score, A: Very high confidence in the interaction, B: High confidence in the interaction, full table with lower confidence scores is included in the supplement.

## Data Availability

The raw data supporting the conclusions of this article will be made available by the authors on request.

## Supplementary Materials

The following supporting information can be downloaded at: https://www.mdpi.com/article/10.3390/jcdd13010019/s1, Table S1: List of antibodies; Table S2: Y2H—Comparative proteomics analysis of Myozap-TG, DesD7-TG and CryABR120G-TG mice; Figure S1: Effects of MLF2 regulation by an adenoviral vector on LC3B, BECN1, LAMP1, RAB7, and ubiquitinylation; Figure S2: Effects of MLF2 regulation on markers of the unfolded protein response; Figure S3: Mlf2 protein levels in different hypertrophy models; Supplementary Data S1: Full Data Co-IP LCMS; Supplementary Data S2: Full Data Y2H.

## Abbreviations

The following abbreviations are used in this manuscript:

Ad	Adenovirus
ARVCM	adult rat ventricular cardiac myocytes
Cn-TG	Calcineurin transgene
CryAB	α-B-crystallin
CryAB^R120G^-tGFP	α-B-crystallin^R120G^-tGFP
CryAB^wt^-tGFP	α-B-crystallin^wt^-tGFP
DCM	dilated cardiomyopathy
DRM	Desmin-related cardiomyopathy
ER	Endoplasmic reticulum
HERPUD1	homocysteine-responsive endoplasmic reticulum-resident ubiquitin-like domain member 1
MFL1	Myeloid leukaemia factor 1
MLF2	Myeloid leukaemia factor 2
NRVCM	neonatal rat ventricular cardiac myocytes
PE	Phenylephrine
TAC	transverse aortic constriction
Y2H	Yeast two-hybrid
