# Supplementary material for: First Glance at Myeloid Leukaemia Factor 2 in Cardiomyocytes
Source: J Cardiovasc Dev Dis. 2025 Dec 30;13(1):19. doi: 10.3390/jcdd13010019 (PMC12841798; doi:10.3390/jcdd13010019)
Supplement: Supplementary file 1 [file jcdd-13-00019-s001.zip › 2025-12-13 MLF2_Supplement_JCDD final.pdf]

Suppl. Table S1: List of antibodies

host-target (target-species)	method	company	Ref. number	dilution
rb-BECN1 (rt)	WB	Cell Signalling	3495S	1:1000
rt-LAMP1 (rt)	WB	Santa Cruz	sc-19992	1:500
rb-RAB7 (rt)	WB	Sigma	R4779	1:1000
rb-LC3B (rt)	WB	Cell Signalling	sc-4108	1:1000
rb-p62 (rt)	WB	Abcam	ab109012	1:10000
ms-α-Tubulin (rt)	WB	Merck	T5168	1:8000
ms-GAPDH (rt)	WB	Sigma	G-8796	1:10000
do-HRP (ms)	WB secondary	Jackson Lab	715-035-150	1:10000
do-HRP (rb)	WB secondary	Jackson Lab	711-035-152	1:10000
do-AF546 (ms)	WB secondary	Invitrogen	A10036	1:2000
ch-AF647 (ms)	WB secondary	Invitrogen	A21463	1:2000
ms-MLF2 (rt/ms) [IgG2b]	WB/IF	Santa Cruz	sc-166874	1:2000/1:100
ms-CryAB (rt) [IgG1]	WB/IF	Abcam	ab13496	1:1000/1:100
ms-HERPUD1 (rt) [IgG2a]	IF	Origene	TA507019	1:100
ms-a-actinin (rt/ms)	IF	Sigma	A7811	1:200
rb-a-actinin (rt/ms)	IF	Abcam	ab137346	1:500
AF488 (rb) for a-actinin	IF secondary	Invitrogen	A21441	1:400
[IgG1] AF594 (ms) for CryAB	IF secondary	Invitrogen	A21125	1:400
[IgG2b] AF647 (ms) for MLF2	IF secondary	Invitrogen	A21242	1:400
[IgG2a] AF546 (ms) for HERPUD1	IF secondary	Invitrogen	A21133	1:400

Suppl. Table S2: Comparative proteomics analysis of Myozap-TG, DesD7-TG and CryAB^{R120G}-TG mice

Sample	Accession	Description	Σ# Proteins	Σ# Unique Peptides	Σ# Peptides	Σ# PSMs
Myozap-TG	Q9QWV4	Myeloid leukemia factor 1	1	6	6	49
	Q99KX1	Myeloid leukemia factor 2	1	6	6	29
DesD7-TG	Q9QWV4	Myeloid leukemia factor 1	1	3	3	11
	Q99KX1	Myeloid leukemia factor 2	1	3	3	11
CryABR120G-TG	Q9QWV4	Myeloid leukemia factor 1	1	4	4	13
	Q99KX1	Myeloid leukemia factor 2	1	6	6	30
WT	Q9QWV4	Myeloid leukemia factor 1	0	0	0	0
	Q99KX1	Myeloid leukemia factor 2	0	0	0	0

Suppl. Table S2: Comparative proteomics analysis of Myozap-TG, DesD7-TG and CryABR120G-TG mice. WT: wild-type, PSM: peptide spectrum match, TG: transgenic

Suppl. Fig. S1:

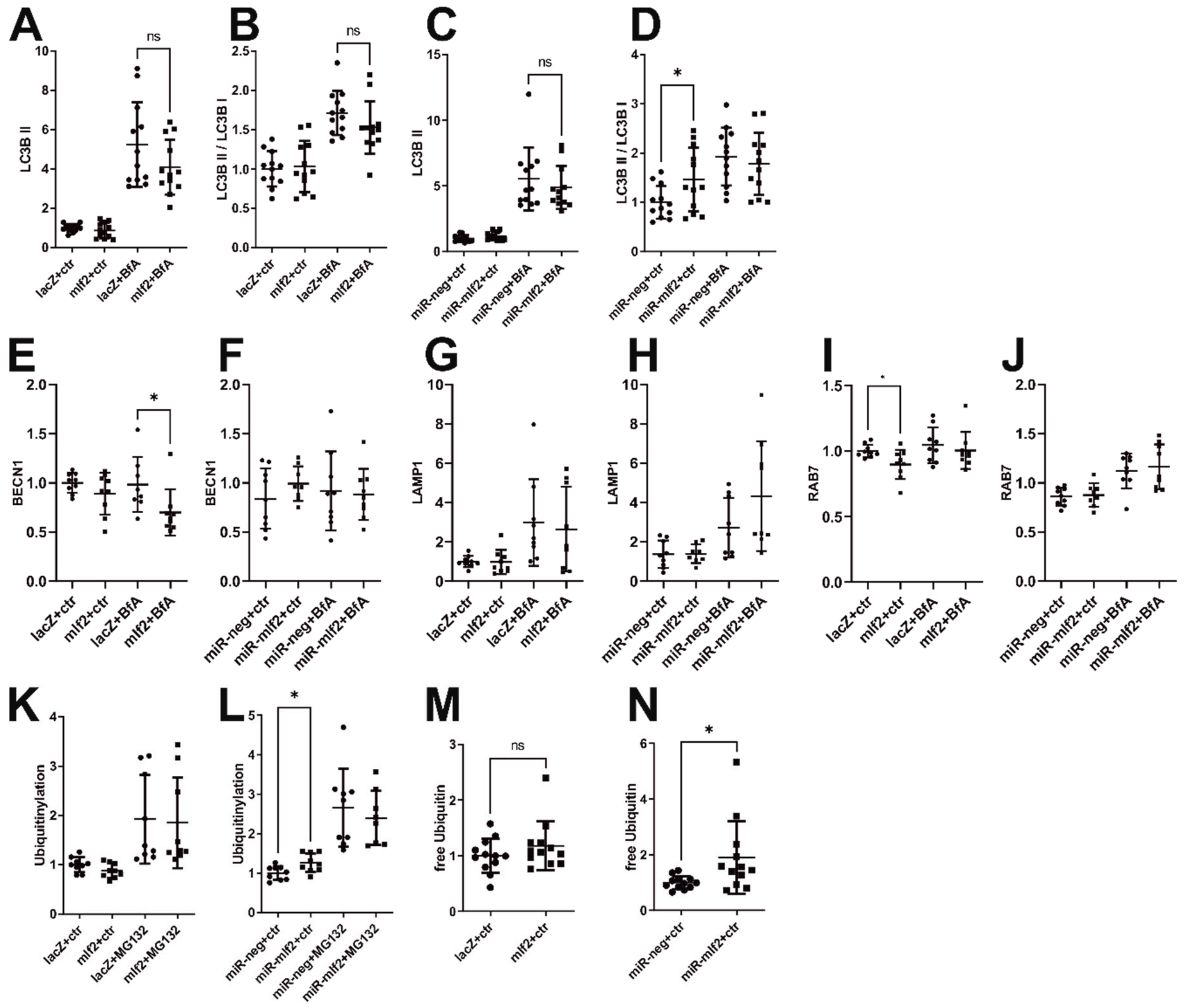

Suppl. Fig. S1 Effects of MLF2 regulation by an adenoviral vector on LC3B **(A-D),** BECN1 **(E & F)**, LAMP1 **(G & H)**, RAB7 **(I & J)**, and ubiquitinylation **(K-N),** NRVCM were treated with Bafilomycin (BfA) or MG132 and infected with Ad MLF2 or Ad miR MLF2 and the respective controls. Protein expression was determined by immunoblotting (followed by quantification by densitometry). Statistical analysis using two-tailed Student's t-test. $*p < 0.05$ ns: not significant

Suppl. Fig. S2:

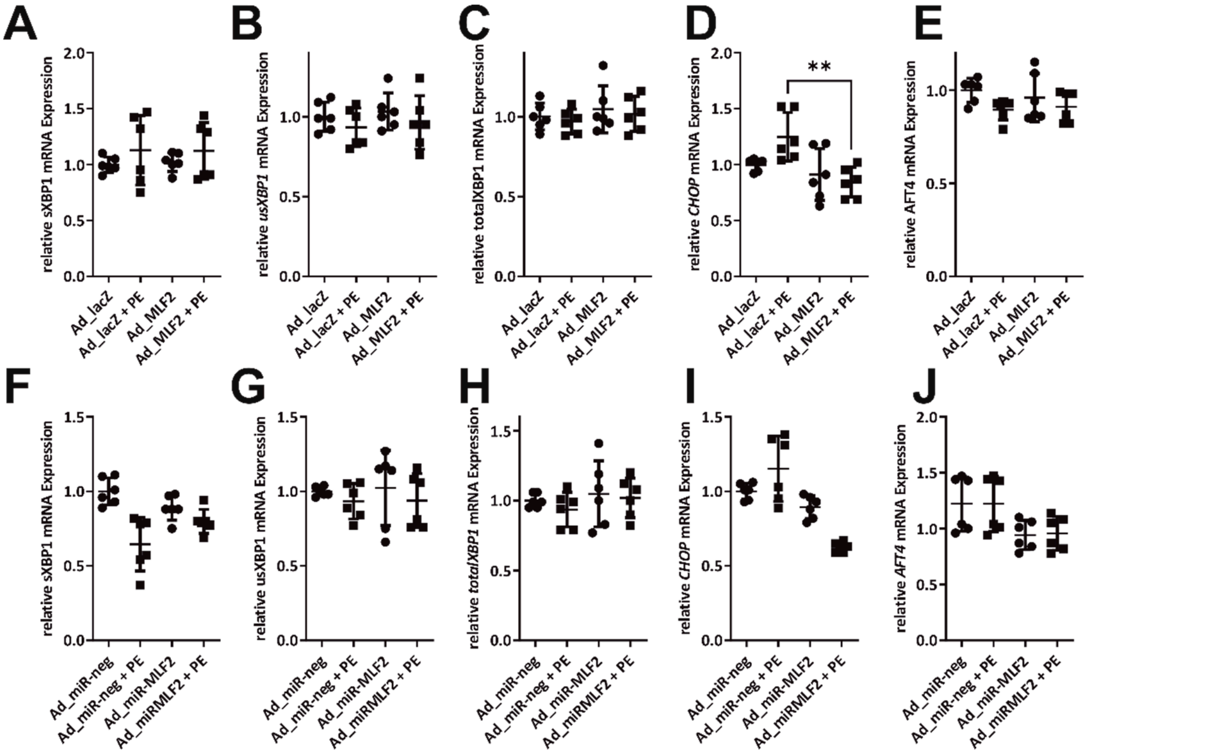

Suppl. Fig. S2 *Effects of MLF2 regulation on markers of the unfolded protein response.* In NRVCM, MLF2 was regulated by adenoviral vectors containing either an overexpression construct or a micro-RNA for knockdown of MLF2. Vectors containing lacZ or miR-neg served as controls. Overexpression of MLF2: upper row, knockdown of MLF2: lower row. Expression of *s-/us-/total Xbp1* **(A-C & F-H)**, *Chop* **(D & I)** and *Atf4* **(E & J)** determined via qRT-PCR. Statistical analysis using two-tailed Student's t-test. $**p < 0.01$

Suppl. Fig. S3:

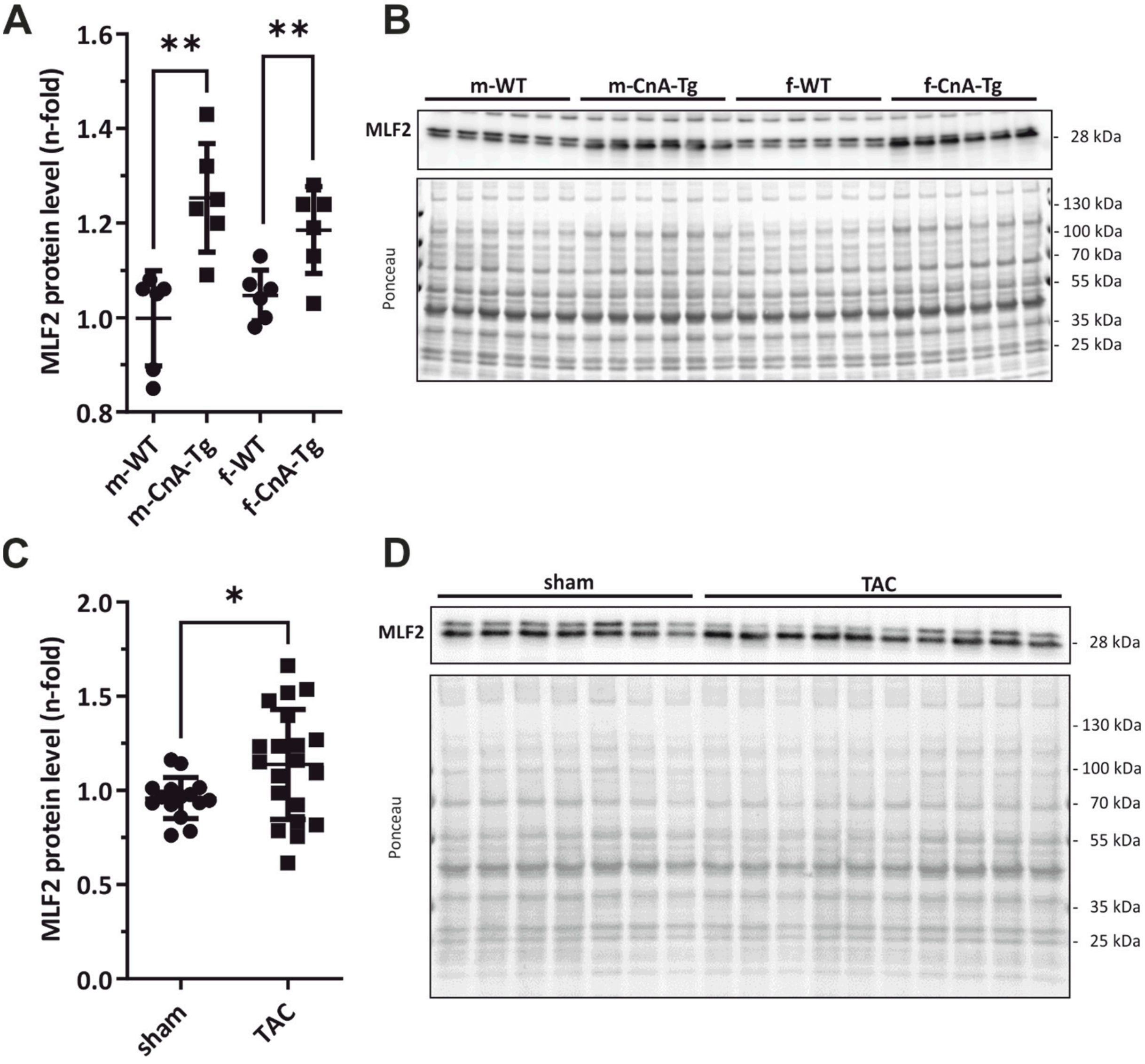

Suppl. Fig. S3 *Mlf2 protein levels in different hypertrophy models: (*A, B) calcineurin-transgenic mice (CnA-TG) versus wild type (WT) controls (by sex: female = f and male = m). (C, D) αMHC-Cre mice after TAC or sham surgery (ROUT (Q=1%): 1 outlier in sham group removed; t-test (p=0.031); t-test including all samples (p=0.114)). Statistical analysis using two-tailed Student's t-test. **p < 0.01
